# The evidence for a role of vasospasm in the pathogenesis of cerebral malaria

**DOI:** 10.1186/s12936-015-0928-4

**Published:** 2015-10-13

**Authors:** Michael Eisenhut

**Affiliations:** Luton and Dunstable University Hospital NHS Foundation Trust, Lewsey Road, Luton, LU4ODZ UK

**Keywords:** Cerebral malaria, Nitric oxide, Haemoxygenase, Vasospasm, Vasoconstriction

## Abstract

Due to delay in treatment, cerebral malaria (CM) remains a significant complication of *Plasmodium falciparum* infection and is a common cause of death from malaria. In addition, more than 10 % of children surviving CM have neurological and long-term cognitive deficits. Understanding the pathogenesis of CM enables design of supportive treatment, reducing neurological morbidity and mortality. Vaso-occlusion and brain swelling appear to be leading to clinical features, neuronal damage and death in CM. It is proposed that parasitized red blood cells (pRBC), due to cytoadhesion to the endothelium and vasospasm induced by reduced bioavailability of nitric oxide, are causes. Stasis of blood flow and accumulation of pRBC may allow, after schizont rupture, for high concentration of products of haemolysis to accumulate, which leads to localized nitric oxide depletion, inducing adhesion molecules and cerebral vasospasm. Features consistent with an involvement of vasospasm are rapid reversibility of neurological symptoms, intermittently increased or absent flow in medium cerebral artery detectable on Doppler ultrasound and hemispheric reversible changes on cerebral magnetic resonance imaging in some patients. Clinical trials of treatment that can rapidly reduce cerebral vasospasm, including nitric oxide donors, inhaled nitric oxide, endothelin or calcium antagonists, or tissue plasminogen activators, are warranted.

## Background

A significant proportion of the 584,000 (95 % uncertainty interval, 367,000–755,000) malaria deaths in 2013 [1] were due to cerebral malaria (CM). The majority of affected children were living in sub-Saharan Africa and were under 5 years old. For clinical purposes, CM has been defined by the WHO as an encephalopathy, where asexual *Plasmodium falciparum* parasitaemia is detectable in peripheral blood and without an alternative cause. Even with optimal care CM was reported to have a mortality of 15–25 % in children and 20 % in adults [2, 3]. Fifty to 75 % of deaths were found to occur within 24 h of admission to hospital [4]. This means any supportive treatment aimed at reducing mortality in CM has to be able to rapidly reverse key pathophysiological events leading to CM. Targeting inflammatory cascades or the sequestered parasites, which have been associated with the pathogenesis of CM, may take effect with a delay of days. Targeting the end result of brain swelling, which is linked to mortality [2] by osmotic therapy, has not been successful [5]. There is, therefore, an urgent need to identify rapidly reversible mechanisms of CM.

Mouse models have been used to investigate potential treatments for human CM. The cerebral pathology in the mouse model of CM (experimental cerebral malaria (ECM)) is fundamentally different from human CM in the sense that in Plasmodium berghei (ANKA strain) infection of C57BL/6 or CBA mice, the CM is characterized by sequestration of leukocytes in the brain, while in human CM mainly parasitized red blood cells (pRBC) are sequestered. Conclusions drawn from findings in this model may not apply to humans [6]. Mice depleted of lymphocytes do not develop white cell sequestration in the brain. Vasospasm has, on the basis of histopathological findings, first been proposed to be involved in human CM by Polder et al. [7] and would be accessible to treatment successfully applied in other diseases associated with cerebral vasospasm, such as sub-arachnoid haemorrhage. This review had the objective to identify and summarize features of cerebral vasospasm in previous studies of human CM and explore how these findings could be used to lay the foundations of therapeutic approaches.

## Review

### Key features of cerebral malaria

Neurological features of acute CM and long-term neurological deficits were comprehensively reviewed previously [4]. Following the WHO definition, an essential feature of CM is coma with the inability to localize painful stimuli. Investigation of brain activity during coma revealed bilateral slowing on electroencephalography. Seizures may occur in up to 60 % of hospitalized children. Seizures are less common in adults where they occur in 20 %. There may be brainstem signs, including abnormal eye movements, changes in pupillary size and reaction, absence of corneal and oculocephalic reflexes, disorders of conjugate gaze and abnormal breathing patterns, abnormal posture, and abnormalities of tone and reflexes. Such signs are more common in children than in adults. Recovery of consciousness occurs usually within 48 h. Features of poor prognosis for survival and long-term morbidity were deep coma, multiple and prolonged seizures and absent corneal and oculocephalic reflexes. Elevated lactate levels in plasma and cerebrospinal fluid were linked to increased mortality.

Electroencephalography during seizures showed that the origin of seizures was often the temporo-parietal region (a watershed area). The review authors suggested that this may indicate that ischaemia and hypoxia are involved in its aetiology.

### Neurological deficits in survivors of cerebral malaria

Neurological deficits in children with CM summarized from previous studies [4] were identifiable in 11 % and include hemiparesis (4.4 %), quadriparesis (3.5 %), ataxia (2.5 %), visual (2.3 %) and speech (2.1 %) impairments, hearing loss (1.9 %) and behavioural difficulties (1.3 %).

In adults neurological deficits as sequelae were observed in less than 5 % and characteristically included extrapyramidal tremor and other cerebellar signs, cranial nerve palsies, mononeuritis multiplex, and polyneuropathy [4]. In the majority of survivors of CM neurological deficits were reversible.

### The result of imaging studies

In a recent study, magnetic resonance imaging (MRI) was performed in 38 paediatric survivors of CM. Abnormalities found were focal cortical defects (16 %), sub-cortical T2 signal changes (18 %), atrophy (47 %), and periventricular T2 signal changes (53 %). Acute papilloedema occurred in patients with sub-cortical T2 signal changes. Periventricular T2 signal changes were found in patients with peripheral retinal whitening [8].

A large, detailed MRI study on 152 children with CM was conducted in Malawi and compared findings in retinopathy-positive and -negative patients; MRI features, which were more common in children with retinopathy were brain swelling, basal ganglia involvement, cortical abnormalities on T2, periventricular white matter changes, brainstem abnormalities on T2, thalamic involvement and corpus callosum changes on T2 and diffusion-weighted images, as well as cerebellar changes. In children with retinopathy-positive CM, it is important to note that some had unilateral hemispheric predominance of oedema, predominance in the globus pallidus and cortical involvement was noted to be “occasionally markedly asymmetrical hemispheric” [9]. In a case of CM reported by another group, single photon emission computed tomography was performed. Focal right hemispheric hypoperfusion was found, which correlated to the patient’s right hemispheric localizing signs. Transcranial dopper sonography however was normal in this patient. Neurological symptoms and abnormalities on imaging subsequently completely resolved [10]. Features of brain swelling were found to be more common in children with CM compared to adults and more common in children, who died of CM [2]. Brain swelling may explain that brainstem signs and papilloedema are more common in children [4, 11].

### In vivo studies of angiopathy in cerebral malaria

#### Retinal changes on fundoscopy and fundus fluorescein angiography

Recent in vivo studies on retinas in 34 Malawian children with CM by fluorescein angiography showed zones of capillary non-perfusion in 76 % of patients, involving the macula in 56 % and the retinal periphery in 65 %, indicating the presence of obstructed retinal capillaries and postcapillary venules. The blood vessels affected were matched by retinal whitening in fundal photography. In 26 % of patients, angiography showed large occluded vessels with resulting ischemic areas in the supplied peripheral retina downstream. There were vessels with abrupt pruning of larger arterioles with normal perfusion right up to the point of non-perfusion. Normal angiograms in six patients, including two with neurological sequelae, indicated a lack of long-standing vaso-occlusion [12].

An observational study was performed in 42 adults with falciparum malaria, of which 27 patients were suffering from complicated malaria. The study used bedside, portable, high-resolution, digital retinal photography and compared patients with complicated and uncomplicated falciparum malaria.

Malarial retinopathy was present in 63 % of adults with severe malaria, in 70 % with CM and in 43 % with non-cerebral severe malaria. The severity of retinal findings correlated with disease severity. Admission blood lactate levels correlated significantly with the severity of retinal and macular whitening in patients with severe malaria. Haematocrit on admission correlated negatively with the quantity of white-centred haemorrhages. The severity of retinal haemorrhages correlated with anaemia. Differences between retinopathy in adults and children evident from this study were that blood vessel discolouration detected in about 30 % of children with CM was not found in any of the adults. The vessel discolouration was compatible with depletion of stationary RBCs infected with mature parasites of haemoglobin.

Retinal whitening and discolouration of retinal blood vessels, but not retinal haemorrhages, were associated with occluded blood vessels on fluorescein angiography [13].

#### Changes of vascular tone

Peripheral arterial tonometry measures the change in digital pulse wave amplitude in reaction to hyperaemia resulting in a reactive hyperaemia peripheral arterial tonometry (RH-PAT) index, which provides a measurement of reactive vasodilation. The arterial pulsatile volume is measured in the index finger at rest and compared with that obtained after an increase induced by 5 min of forearm ischemia (sphygmomanometer inflated to 200 mmHg) and with values obtained in the contralateral index finger without reactive hyperaemia. It is known that the RH-PAT index is partially (≤50 %) reflecting endothelial nitric oxide (NO) production.

Patients with severe malaria had a significantly reduced RH-PAT index in the peripheral circulation compared to uninfected controls or patients with uncomplicated malaria [14].

In another study using RH-PAT, patients with severe malaria had a significantly lower RH-PAT index and cell-free haemoglobin, and plasma arginase levels were inversely correlated to RH-PAT indexes. In patients who survived >24 h after enrolment, there were significant associations between increase in RH-PAT index and both decreasing cell-free haemoglobin and increasing l-arginine concentrations [15].

#### Changes of blood flow on cerebral Doppler studies

Cerebral vasospasm has been defined by Lindegaard using blood velocity measurements by transcranial Doppler ultrasounds. He found that a narrowing of vessel diameter on angiography is associated with increased velocity with a ratio of >3 in middle cerebral artery to extracranial internal carotid artery flow diagnostic of vasospasm [16].

In a pioneering study, Newton et al. investigated cerebral haemodynamics by transcranial Doppler [17]. On investigation of 50 children with CM, in 30 % an increase in cerebral blood flow velocity was observed. Six of 11 children with neurological sequelae had sonographic abnormalities associated with unilateral deficits, including two children where the contralateral middle cerebral artery could not be ultrasonicated and two where seizures were associated with a transient increase in Doppler velocity. In three children, who died, ultrasonographic features of increased intracranial pressure were documented. Subsequent studies confirmed the finding of an increased blood flow velocity in CM [18].

### The role of nitric oxide in cerebral malaria

Over recent years the role of NO in the pathogenesis of CM including reduction of pRBC sequestration and reduction of vasospasm has been increasingly recognized. NO is an endogenous vasodilator produced in endothelium by the endothelial NO synthase (eNOS) enzyme. Without the presence of RBCs NO has a half-life of up to several minutes. It can therefore diffuse across cell membranes and act as an intercellular messenger. NO diffuses from the endothelium to nearby smooth muscle. There it binds to the haem group of the guanylate cyclase. This causes this enzyme to convert guanosine triphosphate (GTP) to cyclic guanosine monophosphate (cGMP) and cGMP then activates cGMP-dependent protein kinases and produces vasodilatation [19].

NO can down-regulate transcription of endothelial adhesion molecule genes, including E-selectin, VCAM-1, ICAM-1 and P-selectin. NO increases the expression of endothelin B receptors (a receptor mediating vasodilatation) and decreases expression of endothelin-1, the most powerful endogenous vasoconstrictor [20].

Inhibition of NO production by exposure of human microvascular endothelial cell to the NOS inhibitor L-N(G)-nitro-arginine-methyl-ester (L-NAME) increased *P. falciparum* pRBC adhesion, while addition of a NO-donor reduced adhesion. *In vitro* studies showed that the NO donor 3-(2-hydroxy-2-nitroso-1-propylhydrazino)-1-propanamine (PPN) treatment reduced the number of adherent pRBCs on resting and TNF stimulated human microvascular endothelial cells (HMECs) by down-regulating basal ICAM-1 expression [21]. NO effects therefore have features which may be protective against complications of *P. falciparum* infection by inhibition of cyto-adherence. Patients with CM were found to have decreased plasma and cerebrospinal levels of the nitric oxide metabolites nitrite and nitrate [22].

Patients with CM also had reduced levels of l-Arginine, the substrate for NO production by nitric oxide synthase. Hypoargininaemia was strongly associated with mortality [23].

### The role of haemolysis in cerebral malaria

**Fig. 1:**
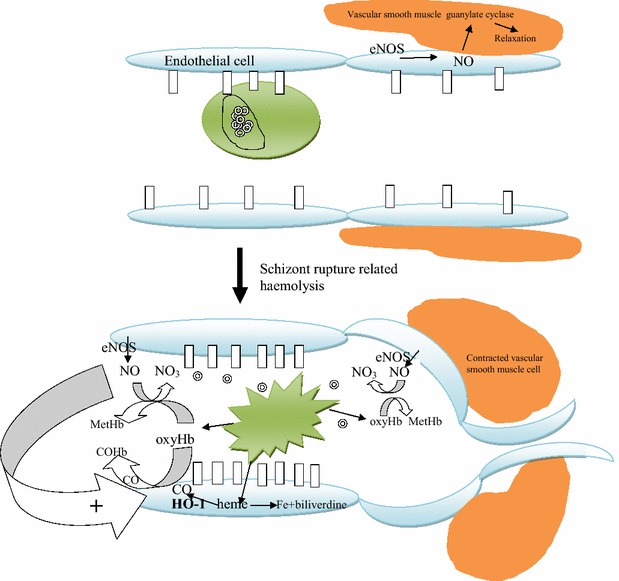
Model for the interaction between sequestration of parasitized red blood cells (pRBC), haemolysis, nitric oxide depletion, and vasospasm. Red blood cells containing schizonts adhere to endothelial cells bound by intercellular adhesion molecule-1 (ICAM-1) symbolized by a vertical rectangle and rupture releasing free haemoglobin (oxyHb), the haem moiety of which binds nitric oxide (NO) generated by endothelial nitrix oxide synthase (eNOS) diverting it from diffusion to vascular smooth muscle cells and converting it to methaemoglobin (MetHb). The vascular smooth muscles depicted, can be distant from the site of sequestration anywhere on the arterial side of the circulation. The depletion of NO causes vasoconstriction and increased expression of ICAM-1. Without oxy haemoglobin nitric oxide activates the vascular smooth muscle guanylate cyclase which generates cGMP which via activation of a protein kinase leads to muscle relaxation. Haemoxygenase (HO-1) metabolizes haem into carbon monoxide (CO), iron and biliverdine. CO binds haemoglobin and prevents binding of nitric oxide and hence nitric oxide metabolism

Replication of schizonts inside RBCs leads to haemolysis and therefore to production of cell-free haemoglobin which after exposure to reactive oxygen species is oxidized. It is established that during haemolysis, cell-free oxyHb in the plasma acts as a NO scavenger leading to production of metHb and nitrate (NO_3_^−^) in a rapid and irreversible reaction [24]. Cell-free plasma haemoglobin has the potential to eliminate the majority of the NO that is generated by endothelial cells [25]. This is counteracted by the process of catabolism of haem released during haemolysis by intracellular haem oxygenase-1 (HO-1), generating carbon monoxide (CO), Fe, and biliverdine. CO has been found to be involved in mediating the protective effect of HO-1. CO hereby binds to the prosthetic haem groups of cell-free haemoglobin leading to the formation of a stable carboxyhaemoglobin complex resistant to oxidizing influences. This reduces the nitric oxide metabolizing capacity of free haemoglobin. The events resulting from haemolysis-associated nitric oxide depletion are summarized in Fig. 1. More recently it was demonstrated that RBC microparticles generated during haemolysis can scavenge NO with a capacity comparable to cell-free haemoglobin [26].

RBC arginase appears in the plasma during haemolysis and is in competition with NOS for the same substrate, l-arginine [27].

Direct evidence for a role of haemolysis in determining vascular tone in severe malaria in humans comes from an observational study in Indonesian adults, which demonstrated that reactive RH-PAT index was correlated with haptoglobin concentrations. Infusion of l-arginine, which is the substrate for the NO synthase producing nitric oxide, significantly increased the RH-PAT index [14].

Support for a role of depletion of nitric oxide by the haemolytic process in the pathogenesis of CM would be an association of elevated free haemoglobin with CM. This has recently been confirmed by comparison of free haemoglobin in children with moderately severe malaria, severe malaria and CM in Tanzania, where only children with CM had a significant elevation of plasma cell free haemoglobin compared to healthy controls [28].

### The role of endothelins

To gather evidence for a role of vasospasm in CM it is essential to examine data on the association of vasoconstrictors with features of CM. Endothelin-1 is one of the most potent vasoconstrictors. Two studies analysing ET-1 during malaria infection in humans have been reported to date [29, 30]. They showed that ET-1 was increased in patients with complicated falciparum malaria compared to controls. There was no correlation between serum levels of ET-1 and severity of disease. Studies conducted in vitro demonstrated that lipid components of the parasite present in the membrane of *P. falciparum*-infected RBCs and malaria pigment (haemozoin) can both bind ET-1 [31]. The authors speculated that non-specific binding could reduce ET-1 activity by decreasing its presence in the local microenvironment where sequestration of pRBCs occurs.

### *Post*-*mortem* examination of patients who died of cerebral malaria

In landmark quantitative analyses of brain sections in *post*-*mortem* studies of patients in Thailand and Vietnam, it was demonstrated that adults with CM had more pRBCs sequestered in the brain than patients who died of other complications of malaria. In addition, pRBC sequestration in the brain of CM patients was also more pronounced compared to other organs [32, 33]. Adults and children succumbing to CM showed significantly increased expression of intercellular adhesion molecule-1 (ICAM-1) in the brain, which mediates adhesion of pRBC to the endothelium of cerebral capillaries and venules [34]. Inflammation involving release of tumour necrosis factor has been involved in up-regulation of ICAM-1 expression in endothelial cells and the process of induction of this inflammation has been reviewed elsewhere [35, 36]. In an autopsy series on children who died of CM, 34 % were found to have no discernible cerebral histological changes, only minimal malarial pigment and very few parasites within cerebral vessels [37]. There was no or little detectable inducible nitric oxide synthase found in cerebral endothelial cells of those patients [38].

#### Retinal histopathology

The most extensive retinal histopathological examination in CM reported so far included findings of the eyes of 64 children, including 13 cases with a clinical diagnosis of CM but an alternative cause of death. All patients with systemic pathological features of CM had prominent sequestration of early or late stage parasites in the retinal vasculature. Haemorrhages were significantly more common in CM patients compared to patients with non-malarial disease. There were significantly more thrombi seen in the retinal vessels of patients with CM than in the non-CM group. Thrombi were found to often only partially occlude blood vessels and seen without surrounding haemorrhage [39].

In a more recent study on the same population in Malawi [40] in 13 children with histologically proven CM, all had malarial retinopathy and showed pRBC sequestration in a mean of 69 % of retinal capillaries and a mean of 81 % of retinal venules. The percentage of pRBC sequestration correlated significantly with the grade of malaria retinopathy severity detected before death. There was a positive correlation of percentage of capillaries with pRBC in the retina and brain. It is important to note that the presence of extra-erythrocytic haemozoin was higher in patients with moderate to severe malarial retinopathy compared to patients with mild malarial retinopathy. The authors noted vascular congestion in retinal venules which showed a high density of pRBCs, confirming a previous histopathological study conducted in Vietnam which demonstrated that congestion of heavily parasitized cerebral micro-vessels was associated with a reduction of Glasgow coma scale (GCS) during coma and shortening of time of coma before death [41].

### Treatment of cerebral malaria

#### Nitric oxide donors

There has been, so far, only been one published trial in humans addressing the bioavailability of the vasodilator nitric oxide. Intravenous infusion of l-Arginine (l-Arg) was associated with an increase in the RH-PAT index and led to an increase in exhaled NO levels in patients with moderately severe malaria [14] and was shown to be safe [42, 43]. In a subsequent, randomized study of intravenous l-Arg infusion in Indonesian adults with severe malaria, no effect on clearance of lactate or RH-PAT index was detected [44]. Currently, randomized controlled trials are awaiting publication [45], or are planned [46] exploring the effect of inhaled nitric oxide as adjuvant treatment of CM.

## Discussion

### The evidence for vasospasm in cerebral malaria

This review shows that previous studies have not demonstrated direct evidence of vasospasm in human CM. Reason for this may be that studies conducted so far have not used internationally recognized criteria, such as the Lindegaard criteria of flow ratio of >3 in middle cerebral artery to extracranial internal carotid artery flow, or cut-offs for definition of significantly increased flow on Doppler ultrasound [16], to define vasospasm in CM. Future studies need to assess patients with this method for evidence of vasospasm. This method previously found evidence for vasospasm in bacterial meningitis where low GCS (<7) on admission, seizures and focal cerebral ischaemic deficits, were more common in patients with cerebral blood flow velocity >210 cm/s, compatible with vasospasm [47].

Data presented above provide the following lines of indirect evidence of cerebral vasospasm in CM:

- The rapid reversibility of coma and neurological deficits in the majority of patients with CM is consistent with a role of transient vasospasm and against a vascular obstruction by thrombi or sequestered pRBCs;
- The unilateral asymmetrical hemispheric changes on cerebral MRI and transient hemispheric changes on single-photon emission computed tomography. described in some patients are compatible with a transient vasospasm in a large supplying artery and cannot be explained by thrombi or sequestration. Obstruction of a large artery by thrombus or sequestered pRBCs has not been found on *post*-*mortem* studies of patients who died of CM;
- Transient increase in Doppler flow during seizures in CM cannot be explained by sequestration or thrombi but by vasospasm. The lack of detection of middle cerebral artery by Doppler in patients associated with transient neurological deficits is also most likely due to transient vasospasm;
- Normal angiograms in six patients, including two with neurological sequelae, indicated a lack of long-standing vaso-occlusion more compatible with vasospasm than other forms of vasocclusion;
- In a sub-group of patients, features of fatal CM were found without sequestration of pRBCs or thrombi in cerebral blood vessels on autopsy. The absence of inducible NO synthase (iNOS) expression in endothelial cells in these patients was against this absence of pRBCs being due to recent simultaneous bursting of sequestered pRBCs, which would have induced iNOS expression;
- RH-PAT shows changes during malaria compatible with changes of nitric oxide bioavailability, which could predispose to and cause vasospasm.

To assess the specific contribution of vasospasm to the pathophysiology of CM, a comparison with a condition characterized by pure and isolated cerebral vasospasm without an underlying inflammation or haemorrhage is helpful. Such a condition is the so-called reversible cerebral vasoconstriction syndrome (see Table 1). The syndrome with a review of all its features and management has been characterized previously [48].The features found in reversible cerebral vasoconstriction syndrome provide evidence that vasospasm causes reversible brain swelling and intracranial haemorrhages, as found in CM.

**Table 1:** Features shared between cerebral malaria and cerebral vasoconstriction syndrome [2, 48]

Clinical features
Seizures, headaches, persistent neurological deficits, including aphasia, hemiplegia, hemianopia, or cortical blindness
Radiological features
MRI findings of sub-arachnoid and intracerebral haemorrhages, cerebral infarction, and reversible brain oedema, hyperintensities on T2 imaging involving the cortex and sub-cortical and deep white matter
Doppler ultrasonographic findings
Maximum mean flow velocities in the middle cerebral arteries might be normal during the first few days after onset of symptoms

Conditions associated with ocular vasospastic syndrome have been associated with retinal arterial vaso-occlusion and ischemia [49]. There is a clear causal link between vasospastic conditions and secondary cerebral haemorrhages as well as evidence for vasospasm being secondary to extravasated blood. In children with CM, haemoglobin depleted retinal blood vessels (secondary to haemolysis) were associated with non-perfusion on angiography and retinal whitening [13] compatible with a role of haemolysis in vaso-occlusion by vasospasm.

### The aetiology of brain swelling in cerebral malaria

Against a disruption of the blood brain barrier as cause of brain swelling in CM, is the fact that radioactively labelled albumin given intravenously was not found in cerebrospinal fluid during coma and no increase was found in the albumin index (ratio of concentrations of albumin in cerebrospinal fluid to those in blood) [50, 51]. Previous investigators proposed a cytotoxic aetiology of brain swelling from ischaemia [4]. Vasospasm could be involved in such ischemia. Hypoxia in vasospasm-induced ischemia can lead to break down of the cytoskeleton which can then lead to a dysregulation of membranous ion transport, which maintains the osmotic gradient across cell membranes and cell layers, and which can prevent cellular and interstitial oedema [52]. The rapid resolution of such oedema in survivors of CM is against a complete obstruction of blood vessels by sequestrated pRBCs or thrombi and is more compatible with a dominance of rapidly reversible vasospasm in its pathogenesis.

Another proposed explanation of brain swelling in CM is pooling of blood in congested cerebral veins from parasite sequestration. Against this hypothesis is the finding of unilateral brain abnormalities on scanning found in some patients with CM, which is unlikely to occur with the disseminated nature of parasite sequestration affecting both cerebral hemispheres.

Cerebral vasospasm is likely to be more reversible than sequestration. Resolution of vasospasm may be an essential requirement for reversal of sequestration by increasing shearing forces by increased flow through widening blood vessels. Vascular resistance is crucially dependent on diameter. The rapid reversibility of vasospasm makes it the ideal target for rapid treatment of this potentially fatal complication of falciparum malaria.

### The potential of treatments of cerebral vasospasm occurring in the context of sub-arachnoid haemorrhages

To find therapeutic options for any vasospasm contributing to the pathogenesis of CM it is essential to be aware of all treatment that has been shown to reduce cerebral vasospasm rapidly in humans and has been demonstrated to be safe in randomized controlled trials. Such trials have been conducted in patients with cerebral vasospasm associated with sub-arachnoid haemorrhages. A similar mechanism to sub-arachnoid haemorrhage-associated vasospasm caused by haemolysis of extravasated RBCs in the sub-arachnoid space may mediate vasospasm through depletion of nitric oxide and induction of endothelin-1 release in CM because of schizont rupture-related haemolysis as well as the breakdown of extravasated blood from the frequently encountered intracerebral microhaemorrhages associated with more severe disease. Treatments that were found to rapidly reverse cerebral vasospasm in sub-arachnoid haemorrhage and have been summarized previously [53] include calcium channel blockers, such as nimodipine or nicardipine, which reduced the risk of poor outcome (dependency, vegetative state or death) and secondary ischemia [54, 55], endothelin receptor antagonists, which reduced the incidence of delayed ischemic neurological deficit (DIND) and angiographic vasospasm [56, 57], and intra-thecal tissue plasminogen activator, which was associated with significant reductions in poor outcomes, DINDs and angiographic vasospasm [58].

The relevance of erythropoietin in CM was demonstrated by the fact that high levels of erythropoietin (EPO) were measured in African children with severe malarial anaemia and were associated with a 80 % reduction in the risk of neurological sequelae during CM [59]. EPO is found in the cerebrospinal fluid only after very high intravenous doses, which can have systemic, including haematopoietic, adverse effects.

It has recently been described that the non-haematopoietic and haematopoietic functions of EPO are transmitted through different receptors. To reduce side effects of EPO, non-haematopoietic EPO derivatives have been generated, such as asialoEpo and carbamylated Epo (CEPO), that maintain their tissue-protective activities but do not increase the concentration of RBCs [60]. EPO has been used to reduce vasospasm in sub-arachnoid haemorrhage in a randomized controlled trial, which was associated with reduced DIND with new cerebral infarcts, a reduction of impaired auto-regulation and better outcome at discharge [61]. There is a need for randomized controlled trials using non-haematopoietic EPO derivatives in treatment of CM.

### Reducing adverse effects of haemolysis

Future research needs to find supportive evidence for the role of haemolysis in CM. This can be gained indirectly by investigating the role of the HO-1 in CM: ferritin, bilirubin and iron could be measured in the cerebrospinal fluid of patients with CM compared to controls with uncomplicated and complicated falciparum malaria, as they are end-products of haem metabolism by HO-1 [62].

Prevention of the effect of free haemoglobin in reducing nitric oxide bioavailability has been achieved by use of haptoglobin [63]. Capturing the nitric oxide scavenging haem group remaining unbound in malaria-induced haemolysis has been achieved by haemopexin (Hx) in other diseases associated with haemolysis [64].

### Inhaled nitric oxide

Inhaled nitric oxide (iNO) could be particularly useful as supportive treatment of CM: unlike nitric oxide donors, such as nitroglycerine and sildenafil, iNO has not been associated with anaemia or systemic vasodilatation causing hypotension. Unlike NO donors, for iNO there is no requirement for a functional endothelial cell NO synthase (eNOS). eNOS may be dysfunctional in some patients. NO gas manufacture is affordable, and can be administered by mask without significant infrastructure requirements [20] and Hawkes et al. are currently exploring its effectiveness in reducing morbidity and mortality in a trial in patients with CM. In addition to developing application of iNO, once effective doses of the phosphodiesterase inhibitor-5 sildenafil, l-arginine, nitrite or nitroglycerin for reducing cerebral vasospasm are established, side effects could be minimized by using combinations of these agents, potentially enabling dose reductions of the individual agent.

## Conclusions

CM remains a significant clinical problem associated with delayed recognition and treatment of falciparum malaria in endemic areas. CM is associated with significant mortality occurring rapidly after admission to hospital. Survival of CM can be associated with permanent neurological and neurocognitive deficits. The key events in the pathophysiology of CM may be vaso-occlusion by sequestration of pRBCs and vasospasm. Haemolysis resulting from rupture of adherent schizonts may cause depletion of nitric oxide by binding of nitric oxide to free haem released by this haemolytic process. Nitric oxide depletion causes vasospasm and increases pRBC adhesion by up-regulation of intercellular adhesion molecule-1 expression. Strategies relieving cerebral vasospasm are most likely to rapidly reverse coma and reduce the risk of ischemic neurological deficit in CM.
